# Mating strategies in *Caenorhabditis elegans* populations are determined by male developmental history

**DOI:** 10.1093/g3journal/jkag146

**Published:** 2026-06-23

**Authors:** Rose S Al-Saadi, Jintao Luo, Alexandra M Nichitean, Nikolaus R Wagner, Nadia G Gaytan, Douglas S Portman, Sarah E Hall

**Affiliations:** Department of Biology, Syracuse University, Syracuse, NY 13244, United States; Department of Biomedical Genetics, University of Rochester, Rochester, NY 14642, United States; Department of Biology, Syracuse University, Syracuse, NY 13244, United States; Department of Biology, Syracuse University, Syracuse, NY 13244, United States; Department of Biology, Syracuse University, Syracuse, NY 13244, United States; Department of Biomedical Genetics, University of Rochester, Rochester, NY 14642, United States; Department of Biology, Syracuse University, Syracuse, NY 13244, United States

**Keywords:** *C. elegans*, pheromone, RNAi, TGF-β, mating, postdauer

## Abstract

Mating strategies, whether sexual or asexual, confer unique costs and benefits to populations and species that facilitate evolutionary processes. In wild isolates of *Caenorhabditis elegans*, mating strategies are dependent on developmental history. Outcrossing significantly increases when one or both parents have transiently passed through the stress-resistant dauer diapause stage. However, the mechanisms of how life history alters mating strategies have not been systematically explored. Sex-specific responses to pheromones are a major driver of mating behaviors in *C. elegans*. We demonstrated previously that postdauer hermaphrodites exhibit a decreased avoidance of the pheromone ascr#3 due to the downregulation of the *osm-9* TRPV channel gene in postdauer ADL neurons. Thus, we hypothesized that altered responses to pheromones in postdauer animals contribute to increased outcrossing. We conducted mating assays using wild type N2 Bristol, as well as *osm-9*, *daf-3/*co*-*SMAD, and *mut-16/*Mutator mutant strains that regulate the activity of hermaphrodite ADL neurons. First, we show that the outcrossing level of N2 Bristol correlated with the developmental history of males, and that postdauer males exhibited an increased ability to detect mates via pheromones compared to control males. In addition, DAF-3 plays a critical role in postdauer males to regulate mating, while playing a more minor role in hermaphrodites. Furthermore, *mut-16* adults exhibited decreased outcrossing, and attempts to rescue the outcrossing phenotype resulted in transgenerational sterility due to defects in hermaphrodite and male germlines. Together, our results suggest a model whereby mating strategy is driven by male developmental history under combinatorial control of TGF-β and RNAi pathways.

## Introduction

Mating strategies, whether sexual or asexual, have both costs and benefits to populations that allow for evolutionary processes to occur. The theoretical cost of sex and its disadvantages as a mode of reproduction are far higher than that of asexual reproduction (Otto 2009). Indeed, sexual reproduction has a 2-fold cost associated with the production of males due to having to share half of the offspring's genome with a mate (Otto 2009). In addition, sexual reproduction deprives individuals of reproductive assurance and increases the risk of predation (Lehtonen et al. 2012; Meirmans et al. 2012). Despite these disadvantages, most reproducing organisms utilize sexual reproduction to some degree. The main advantage that allowed sex to evolve and persist as the major mode of reproduction is meiotic recombination, which generates allelic diversity, allowing for the fixation of beneficial genetic variants and purging deleterious mutations (Weismann 1889; Burt 2000; Whitlock 2000; Agrawal 2001; Siller 2001; Paland and Lynch 2006; Whitlock and Agrawal 2009). Self-fertilization, a form of sexual reproduction, confers the benefits of meiosis and provides reproductive assurance while reducing the 2-fold cost of males (Smith 1978; Charlesworth 1980; Theologidis et al. 2014). Whether sexual reproduction, or outcrossing, is more favorable than self-fertilization depends largely on the interplay between the benefits and the costs of different reproductive modes in a particular environment.

To capitalize on the benefits of multiple mating strategies (outcrossing, self-fertilization, asexual reproduction), some species evolved to use mixed-mating strategies. For instance, daphnids can switch from asexual to sexual reproduction in response to environmental stresses such as low temperature or starvation (LeBlanc and Medlock 2015). Similarly, pea aphids produce wingless females during favorable conditions, then switch to producing winged, sexual morphs in conditions of low food and high population density (Ogawa and Miura 2014). In addition, some plants can regulate their rate of self-fertilization based on environmental conditions, including temperature, light, nutrients, and water availability (Johnson 1971; Campbell and Linskens 1984; Levin 2012). In nematodes, the *Rhabditis* species SB347 is trioecious and can reproduce via mating between males and females in favorable conditions, but develops as self-fertilizing hermaphrodite in unfavorable conditions (Félix 2004; Chaudhuri et al. 2011). However, the molecular mechanisms underlying these changes in mating strategies based on environmental conditions are not well understood.

*Caenorhabditis elegans* nematodes represent an excellent model to investigate such processes, as they exhibit changes in their mating strategies based on environmental experience. *C. elegans* is an androdioecious species where the populations are composed primarily of self-fertilizing hermaphrodites, with rare males arising through either X-chromosome nondisjunction or through outcrossing (Nigon 1949; Hodgkin et al. 1979). In conditions favorable for growth, *C. elegans* will develop continuously through 4 larval stages before becoming reproductive adults (control adults or CON). However, if animals experience unfavorable conditions such as starvation, high temperature, or crowding after hatching, they can enter the stress-resistant larval diapause state called dauer (Cassada and Russell 1975). Once conditions improve, dauer larvae resume development to become reproductive adults (postdauers or PD) (Cassada and Russell 1975; Golden and Riddle 1982). Passage through dauer over successive generations in wild isolates of *C. elegans*, CB4856 and JU440, significantly increased the proportion of males in the population and the level of outcrossing between hermaphrodites and males (Morran et al. 2009). Control and postdauer adults exhibit different transcriptional profiles that underlie altered behaviors and life history traits (Hall et al. 2010; Sims et al. 2016; Ow et al. 2018, 2021, 2024; Webster et al. 2018; Bhattacharya et al. 2019). These altered behaviors could underlie the changes in mating strategies in postdauer animals and promote outcrossing in a typically self-fertilizing population.

*C. elegans* animals secrete an array of pheromone molecules which can trigger a variety of behaviors and developmental decisions such as dauer entry, aggregation, reproductive development, and mating (Golden and Riddle 1982; Macosko et al. 2009). Hermaphrodites produce a blend of ascaroside (ascr) molecules, including ascr#2, ascr#3, and ascr#4, which act synergistically to make up the pheromone “mating signal” at low concentrations (Srinivasan et al. 2008). These pheromones can elicit sexually dimorphic responses such that hermaphrodites and males exhibit avoidance or attraction behaviors, respectively, in response to high concentrations of ascr#3 (Simon and Sternberg 2002; Srinivasan et al. 2008). Acute avoidance of ascr#3 in hermaphrodites occurs via the ADL sensory neurons that connect with the backward movement neurons, AVA and AVD, via chemical synapses (Jang et al. 2012). In contrast, males possess a modified hermaphrodite circuit that suppresses the ADL-mediated avoidance and, depending on the specific assay performed, promotes ascr#3 attraction through the male-specific CEM neurons and the ASK or ADF sensory neurons (Srinivasan et al. 2008; Narayan et al. 2016; Fagan et al. 2018).

Some of these sex-specific behavioral responses have been shown to depend on the expression of the OSM-9 and OCR-2 TRPV channels, which act in the signal transduction pathway downstream of pheromone receptors in amphid sensory neurons (Bargmann 2006; Jang et al. 2012). The expression of the *osm-9* TRPV gene in ADL neurons of hermaphrodites is dependent upon developmental history (Sims et al. 2016; Ow et al. 2024). In postdauer hermaphrodites, *osm-9* is significantly downregulated in ADL neurons, resulting in decreased avoidance in response to high concentrations of ascr#3. The altered expression of *osm-9* in postdauer hermaphrodites is regulated transcriptionally through a novel regulatory sequence called the “MSP motif” (Ow et al. 2024). We demonstrated previously that the DAF-3/co-SMAD in the TGF-β signaling pathway and the ZFP-1/AF10 chromatin binding protein bind to the *osm-9* MSP motif in postdauer ADL neurons to promote its downregulation. Interestingly, RNAi pathways are required for DAF-3 and ZFP-1 to bind at the *osm-9* promoter, suggesting that RNAi may trigger its downregulation after dauer (Sims et al. 2016).

Given that mating is facilitated by pheromone detection, we tested the hypothesis that sex-specific altered responses to pheromones due to altered *osm-9* expression promote changes in mating strategies in *C. elegans* postdauer populations. Here, we show that the developmental history of males is the primary driver of mating levels, with a minor role for the developmental history of hermaphrodites. We found that postdauer males do not have altered expression of *osm-9* in ADL neurons, but they do exhibit an increased ability to locate mates via pheromones compared to control males. In addition, DAF-3/co-SMAD is required in both males and hermaphrodites to regulate outcrossing levels following dauer. Similarly, we found that RNAi protein MUT-16 is also required for increased mating in postdauer populations. Attempts to rescue the MUT-16 function in neurons resulted in germline defects in hermaphrodites and males, leading to transgenerational sterility. Together, our results show that *C. elegans* animals engage multiple mechanisms to increase the outcrossing efficiency in postdauer populations.

## Materials and methods

### Strain maintenance

The strains used in this study were grown on standard NGM plates seeded with *Escherichia coli*  OP50 and cultivated at 20 °C unless specified otherwise (see Reagent table, Supplementary Table 1). *mut-16(pk710)* and neuronal overexpression strains were maintained at 15 °C due to the temperature-sensitive mortal germline phenotype. Details of strains, genes, and alleles was obtained, in part, through the Alliance of Genome Resources (Alliance of Genome Resources Consortium 2024).

Control animals were maintained on plates where they had plentiful food and low population density for at least 3 generations. Dauer was induced by growing an age-mixed population on 60 mm NGM plates until food depletion. Approximately 3–5 d later, plates were visually examined for dauers, then washed with 1% SDS to isolate the dauer larvae (Karp 2018). The mutant strain *daf-3(mgDf90)* exhibits a dauer-deficient phenotype and was grown on 100 mm NGM plates seeded with concentrated bacteria (20× OP50) at 25 °C for a week to induce dauer formation, followed by a wash with 1% SDS to isolate the dauer larva (Sims et al. 2016; Bharadwaj and Hall 2017). All dauer larvae were rescued on NGM plates with food and maintained at 20 °C.

### *osm-9(0)* strain construction

The *osm-9* coding sequence was deleted from the wild-type genome using the co-CRISPR method as previously described (Arribere et al. 2014; Paix et al. 2015). sgRNAs targeting the *osm-9* locus were designed using the IDT CRISPR-Cas9 guide RNA design checker and cloned into pDD162 using site-directed mutagenesis PCR. An injection mix was made for use with wildtype adults with 25 ng/uL each of *osm-9* sgRNA, *dpy-10* sgRNA, and *dpy-10* repair template. Individual hermaphrodites from jackpot broods were used to create clonal populations and genotyped for the *osm-9* deletion. A line positive for the *osm-9* deletion was backcrossed to remove the *dpy-10* mutation and verified to be homozygous for the desired mutation. The deletion breakpoints are GGGTG 372 bp upstream of the start codon and GAAGT 307 bp downstream of the stop codon. This protocol established the SEH272 strain, henceforth refered to as *osm-9(0)* (Supplementary Table 1).

To rescue *osm-9* expression in ADL neurons in the *osm-9(0)* strain, we crossed the *osm-9* rescue transgene from SEH230 *osm-9(ky10)*; *pdrEx29*[*sre-1*p::*osm-9*::*gfp; unc-122*p::*dsRed]* strain into the *osm-9(0)* strain. The final strain, SEH371 *osm-9(pdr3)*; *pdrEx29*, was genotyped and verified by Sanger sequencing. Primer sequences used for strain creation and genotyping are available upon request.

### *mut-16* overexpression strains

The *mut-16* expression transgene, driven by the *rab-3*, *sre-1*, *gpa-4*, and *srh-142* promoters were maintained as an extrachromosomal array by selecting for animals that exhibit the *unc-122*p::*dsRed* (*array+*) coinjection marker as described in Bharadwaj and Hall (2017). Due to low transmission rates of the extrachromosomal transgene, we integrated the transgene array into the genome following a UV-irradiation protocol for the pan-neuronal (*rab-3*p) and ASI (*gpa-4*p) *mut-16* overexpression strains (Evans 2006). The SEH378 strain was made by injecting germlines of N2 Bristol hermaphrodites with the *rab-3*p::*mut-16*::*gfp* and *unc-122*p::*dsRed* plasmids together at 8 ng/µL and 30 ng/µL, respectively, and selecting for *array+* progeny.

### Mating assays

Mating assays were conducted in the following combinations of continuously developed control (CON) and postdauer (PD) adult males (M) and hermaphrodites (H): CON-H × CON-M, CON-H × PD-M, PD-H × CON-M, and PD-H × PD-M. For most assays, 6 L4 stage hermaphrodites and 6 young adult males were allowed to mate on a single plate (Supplementary Table 2). The parents were placed on 60 mm NGM plates and allowed to mate for 23 h, followed by the removal of males from the plates. Hermaphrodites were then placed on new plates daily until egg-laying ceased and the number of progeny (hermaphrodites and males) per assay was determined. At least 3 mating assays using biologically independent populations were performed for each condition.

The mating efficiency was determined using the proportion of males in the F1 population after mating. Since males are produced through either mating or nondisjunction events of the X-chromosome, the mating efficiency can be determined by subtracting the frequency of X-chromosome nondisjunction in that strain from the overall observed male frequency ($\mathrm{mating}\;\mathrm{efficiency}=\frac{number\;of\;male\;progeny}{number\;of\;total\;progeny}-ChrX\;nondisjunction\;frequency$). To determine the X-chromosome nondisjunction frequencies, we conducted brood size assays using at least 3 biological replicates of each genotype, with 6 hermaphrodites tested for each trial. The proportion of males in the progeny of each trial was averaged across replicates (Supplementary Fig. 1; Supplementary Table 3). There was no significant difference between CON and PD male frequencies; thus, CON male frequency values were subtracted from either condition. The value for N2 male frequency is ≤0.002 as reported by Teotónio et al. (2006). To compare the mating assay results of the different conditions, a two-way ANOVA was performed for assays within a genotype. Multiple unpaired *t*-tests with FDR correction were used to detect significant male frequency differences within a condition. All statistical tests were performed using GraphPad Prism version 10.2.0 for macOS, GraphPad Software, San Diego, California USA.

### Measuring *osm-9* expression levels

The expression levels of *osm-9* were measured using an integrated transgene array expressing GFP under the regulation of 350 bp of *osm-9* upstream regulatory sequences (*pdrIs1*) as previously described (Sims et al. 2016). The expression of GFP was determined in control and postdauer adults for wild-type and *mut-16(mg461)* strains 24 h after the L4 larval stage. Animals were dye-filled using DiD to identify the location of ADL and mounted on agar pads with sodium azide. GFP expression was visualized using a Leica DM5500B microscope with a Hamamatsu 741 camera controller C10600 ORCA-R2. At least 44 animals over a minimum of 3 biologically independent trials were examined for each condition and genotype. Statistical analysis was performed using multiple *t*-tests followed by Holm-Sidak correction within GraphPad Prism (version 10.2.0).

### Pheromone avoidance assays

**Fig. 1.**
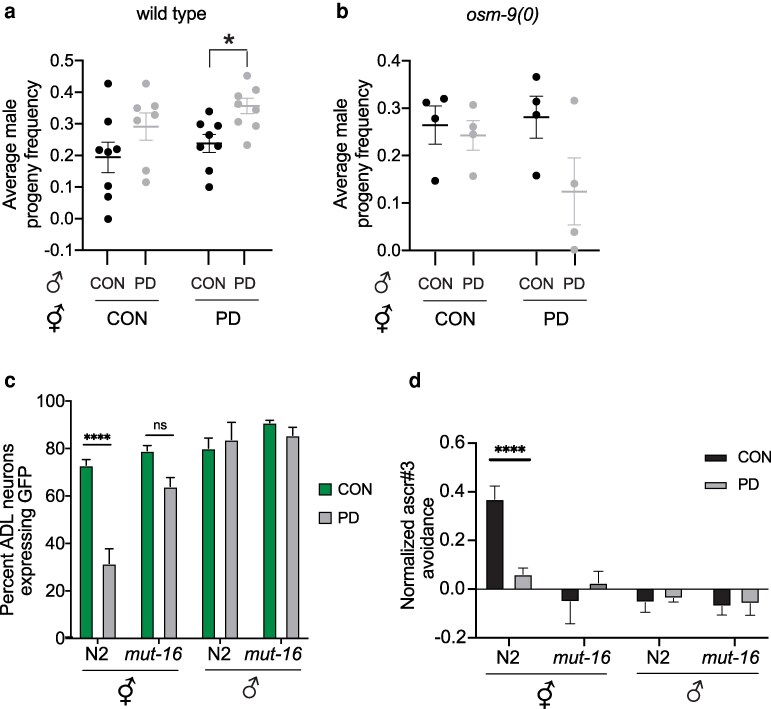
Outcrossing levels in N2 Bristol are dependent upon the developmental history of the males. a, b) Scatter plots with lines representing mean ± S.E.M. male progeny frequencies resulting from mating assays including combinations of CON and PD adults for (a) wild type N2 and (b) *osm-9(0)* strains. * *q* < 0.05, multiple unpaired *t*-tests with FDR correction. Additional data are provided in Supplementary Tables 2 and 4. c) Mean percentage ± S.E.M. of ADL neurons expressing GFP in N2 and *mut-16*(*mg461*) strains. *n* ≥ 44 animals from at least 3 independent trials. **** *P* < 0.0001, multiple *t*-tests followed by Holm-Sidak correction. n.s., not significant. d) Normalized mean ascr#3 avoidance ± S.E.M. for hermaphrodites and males in N2 and *mut-16(pk710)* strains. *n* ≥ 93 animals from at least 3 independent trials. **** *P* < 0.0001, multiple *t*-tests followed by Holm–Sidak correction. Additional data are provided in Supplementary Table 5.

One-day-old adult, control and postdauer males and hermaphrodites were tested for avoidance behavior in response to 100 nM ascr#3 as previously described (Hilliard et al. 2002; Jang et al. 2012; Sims et al. 2016). Avoidance of 1 M glycerol and M13 buffer was tested as the positive and negative controls, respectively. Briefly, animals were picked onto a 60 mm NGM plate without food and allowed to acclimate for 10 min. The drop test acute avoidance response assay was performed using a mouth pipette with a pulled glass needle. An avoidance response was recorded when a forward-crawling animal reversed within 2 s after being exposed to the stimulus. In Fig. 1d, the proportion of animals responding to ascr#3 was normalized by subtracting the proportion of animals responding to the negative control (M13 buffer) as previously described (Hilliard et al. 2002; Jang et al. 2012; Sims et al. 2016). In Supplementary Fig. 2b, ascr#3 response is presented as raw data. Statistical analysis was performed using multiple *t*-tests followed by Holm–Sidak correction or a one-way ANOVA with Tukey's multiple comparisons test within GraphPad Prism (version 10.2.0).

### Brood size assays

For the brood size assays, larval L4 hermaphrodites were placed on seeded NGM plates and transferred to new plates daily until egg-laying ceased, and the number of progeny per plate was determined. For the transgenerational brood size assay, the *mut-16* expression transgene was injected into *mut-16(pk710)* and N2 strains independently, and strains were propagated for more than 10 generations before being used in experiments. A red fluorescent co-injection marker that is easily visualized was used when generating the *mut-16* overexpression strains; thus, *array+* refers to progeny that inherited the extrachromosomal *mut-16/dsRed* transgene array and *array−* refers to the animals that did not inherit the array (Bharadwaj and Hall 2017). Ten hermaphrodites carrying the extrachromosomal array overexpressing *mut-16* pan-neuronally were used to establish the parent plate (P_0_). Ten hermaphrodite progeny (either *array+* or *array−*) were then transferred to a new NGM plate to establish F1 and subsequent generations, and an additional 10 individual hermaphrodites were used to calculate the average brood size. Statistical analysis was performed using multiple *t*-tests followed by Holm–Sidak correction within GraphPad Prism (version 10.2.0).

### DAPI staining

A standard DAPI staining protocol for whole animals was used to visualize germ cell nuclei (Francis et al. 1995; Qiao et al. 1995). Twenty-four hours after the L4 larval stage, animals were fixed in 100% cold methanol, washed with 0.1% PBST, and DAPI-stained using a 1:1000 dilution. Animals were mounted on glass slides and imaged using a Leica DM5500B microscope with a Hamamatsu 741 camera controller C10600 ORCA-R2.

### Germline morphology scoring

The whole-mount DAPI-stained images of each gonad were visually scored based on several criteria, with animals given a score of zero or one based on the absence or presence of each criterion. The different categories were as follows: distal tip cell (DTC) migration defects, egg-laying defects, low gamete number, abnormal gonad morphology, and germline defects. Animals scored for the presence of DTC migration defects include weak to severe meandering on dorsal pathfinding defects (Wong and Schwarzbauer 2012). The one-day-old adult hermaphrodites were scored for potentially having an egg-laying defect if they had a greater number of eggs retained within the uterus in a disorganized manner at the time of staining compared to wild type (Soto et al. 2002). Animals were scored as having a gamete number defect by visually comparing the amount of sperm present between wild-type and mutant animals, which included low to no sperm present. Within the abnormal gonad morphology category, animals were marked for having a smaller overall gonad size and a thinner appearance of the distal gonad compared to wild-type nematodes. Germline defects were quantified by counting the total germ cell nuclei for each gonad arm using the multi-point tool in ImageJ (NIH; Schneider et al. 2012). The germ cell nuclei were further classified into mitotic, transition, and pachytene zones by identifying the characteristic germ cell morphology. The transition and pachytene zones were deemed to start when at least 2 cells in a row showed the crescent-shaped and basket-shaped nuclei morphology, respectively (Shakes et al. 2009). Three trials of biologically independent populations were examined. Statistical analysis was performed using a one-way ANOVA followed by Tukey's multiple comparisons test within GraphPad Prism (version 10.2.0).

### Quadrant mate preference and chemotaxis assays

Mate preference assays were conducted in a *him-5* background to provide sufficient males for testing (Luo et al. 2024). To test whether males have heightened attraction to hermaphrodites following dauer diapause, CON and PD males were used as the tester animals and immobilized hermaphrodites were used as targets. Each quadrant contained 4 target animals, with each quadrant alternating between the *unc-54; him-5* and *unc-54; daf-22; him-5* strains. Ten tester animals were placed in the center of the plate and allowed to move freely about the different quadrants. The location of the tester animals was noted every 30 min for up to 90 min, and the mate preference index was then calculated as previously described (Fagan et al. 2018; Luo et al. 2024). A total of 180 tester animals over 18 trials were examined for this assay. To test if hermaphrodites had an increased attraction to postdauer males, the quadrant assay was used with CON or PD *unc-54; him-5* males alternating as the targets. CON hermaphrodites were used as the tester animals. A total of 160 animals over 16 trials were tested.

To examine ascr#3 attraction, the quadrant assay was performed using ascr#3 and ethanol diluted in water as the alternating targets, and CON and PD males as the testers, largely as previously described (Fagan et al. 2018; Luo et al. 2024). The concentrations of ascr#3 tested, 100 and 10 nM, were chosen to evoke a weak response in control males and not limit the responses of postdauer males due to ceiling effects. The chemotaxis index was calculated by subtracting the number of animals in the control quadrants from the ascr#3 quadrants and dividing by the total number of animals in all quadrants ($\mathrm{chemotaxis}\;\mathrm{index}=\frac{ascr\#\;3-EtOH}{ascr\#\;3+EtOH}$). A total of 100 animals over 10 trials were tested in this assay. Statistical analysis was performed using unpaired *t*-tests within GraphPad Prism (version 10.2.0).

## Results

### Outcrossing levels in N2 Bristol populations are dependent on the developmental history of males

To investigate the molecular mechanisms underlying increased outcrossing in postdauer populations, we first investigated whether the common *C. elegans* lab strain, N2 Bristol, exhibits an increased outcrossing efficiency after passage through dauer as observed previously for other wild isolates, CB4856 and JU440 (Morran et al. 2009). We performed mating assays with all combinations of CON and PD, male and hermaphrodite N2 Bristol adults. For mating assays including CON hermaphrodites and CON males, we observed an average mating efficiency of 0.194 ± 0.048, serving as the baseline of outcrossing levels (Fig. 1a). When either sex experienced dauer (PD males or PD hermaphrodites), we observed a slight increase in outcrossing levels compared to the baseline (Fig. 1a). PD hermaphrodites mated with CON males exhibited a male frequency of 0.238 ± 0.028, while PD Males mated with CON hermaphrodites led to a slightly larger increase in mating success (0.291 ± 0.043). However, when both sexes had experienced dauer (PD males and PD hermaphrodites), we observed the largest increase in outcrossing (0.348 ± 0.025) compared to the mating assays using CON adults (Fig. 1a). Using a two-way ANOVA, we determined that the type of males (CON or PD), but not the type of hermaphrodites, had a significant effect on outcrossing levels in N2 Bristol (*P* = 0.0095) (Supplementary Table 4). We next asked whether male frequencies resulting from mating assays including PD males were significantly greater than those with CON males when the hermaphrodite condition remained constant. For PD hermaphrodites, assays that included PD males showed significantly higher male frequencies than assays with CON males (*q* = 0.011), whereas no significant difference was detected with CON hermaphrodites and CON or PD males (*q* = 0.085). Together, these results demonstrate that postdauer populations of N2 Bristol exhibit increased outcrossing levels similar to the CB4856 (Hawaiian) isolates reported previously and that the developmental history of males is the main determinant of outcrossing levels.

### Expression of *osm-9* is regulated in a sex-specific manner

To test whether the increased outcrossing phenotype in postdauer populations was dependent on the differential expression of OSM-9, we performed the same mating assays to measure outcrossing rates with CON and PD hermaphrodites and males in an *osm-9(0)* mutant strain. We found that all matings yielded similar mating efficiencies (ranging from 0.243 to 0.281), except for the assay where both parents experienced dauer, which showed a slightly decreased mating efficiency (0.124 ± 0.07) (Fig. 1b). The baseline mating level (CON × CON) in the *osm-9(0)* strain (0.264 ± 0.04) is notably higher than observed in wild type (0.194 ± 0.048), suggesting that OSM-9 function may suppress mating (Fig. 1a and b). Additionally, the developmental history of the males or the hermaphrodites was not a significant factor in mating efficiency in the *osm-9(0)* strain. These results indicate that OSM-9 is required for the increased mating in postdauer populations.

To further explore the role of OSM-9 in mating efficiency, we next examined the regulation of *osm-9* in males. Using a strain carrying an integrated GFP reporter transgene driven by ∼350 bp of the *osm-9* promoter (*osm-9*p*::gfp*), we quantified the number of ADL neurons that express GFP in control and postdauer wild-type hermaphrodites and males. As expected, we observed that significantly fewer postdauer hermaphrodites have *osm-9*p::*gfp* expression in ADL compared to controls (Fig. 1c)(Sims et al. 2016). However, in males, GFP is expressed similarly in control and postdauer adult ADL neurons, indicating that regulation of *osm-9* in ADL neurons by developmental history is sex-specific (Fig. 1c). We showed previously that hermaphrodites carrying a mutation in *mut-16* exhibited increased expression of GFP in postdauer hermaphrodite ADL neurons compared to wild type (Sims et al. 2016). MUT-16 is a glutamine/asparagine motif-rich protein that is required for the formation of the *Mutator* focus, a phase-separated perinuclear condensate important for small interfering RNA (siRNA) amplification (Zhang et al. 2011; Phillips et al. 2012; Uebel et al. 2018). To examine if MUT-16 also plays a role in the regulation of *osm-9* expression in males, we examined the GFP expression in ADL neurons in control and postdauer males in the *mut-16* strains. We observed that GFP continued to be expressed in control and postdauer male in the *mut-16* strain, similar to wild type (Fig. 1c), indicating that MUT-16 does not play a role in regulating *osm-9* expression in male ADL neurons.

Acute avoidance of ascr#3 is an ADL-mediated, *osm-9* dependent behavior in hermaphrodites (Jang et al. 2012). We demonstrated previously that postdauer hermaphrodites fail to avoid high concentrations of ascr#3 due to the downregulation of *osm-9* in their ADL neurons (Sims et al. 2016). To examine the ascr#3 avoidance behavior in postdauer males, we tested the response of wild-type and *mut-16* control and postdauer hermaphrodites and males to 100 nM ascr#3 using a drop test assay, as well as M13 buffer (negative control) and 1 M glycerol (positive control) (Fig. 1d; Supplementary Fig. 3). While the positive and negative controls elicited the expected behaviors in both control and postdauer males for the N2 and *mut-16(pk710)* strains (Supplementary Fig. 3), there was no difference in the avoidance behavior in postdauer males compared to control males when exposed to ascr#3 (Fig. 1d). Together, these results indicate that the differential regulation of *osm-9* in ADL neurons due to passage through the dauer stage is hermaphrodite-specific and is unlikely to be a significant driver of outcrossing in postdauer males.

To examine whether OSM-9 was required specifically in male ADL neurons for the increased mating efficiency in postdauers, we expressed wild-type *osm-9* cDNA under an ADL-specific promoter (*sre-1*p) in the *osm-9(0)* strain and performed the mating assays as described above. Again, we observed no discernible increase in male frequency in assays that include postdauer males, similar to the *osm-9(0)* mutant strain (Supplementary Fig. 2a). This observation may be due to the weak rescue of OSM-9-dependent function in ADL (Supplementary Fig. 2b), or that the increased mating phenotype may be due to OSM-9 function in other sensory neurons. Although our original hypothesis that the differential expression of *osm-9* in hermaphrodite ADL neurons promotes outcrossing is not supported by our data, the results presented here indicate that OSM-9 is required for the differences in mating efficiency following dauer diapause.

### Endogenous RNAi regulates mating efficiency

**Fig. 2.**
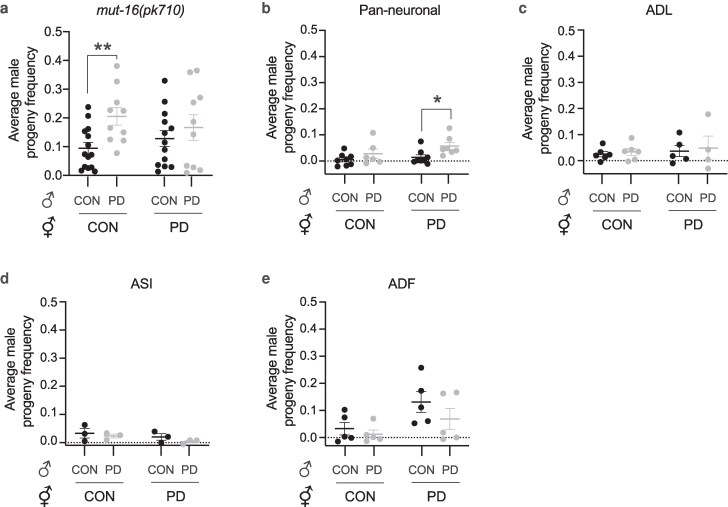
Endogenous RNAi is required for increased outcrossing in postdauer populations. Scatterplots with lines representing mean ± S.E.M. male progeny frequencies resulting from mating assays including combinations of CON and PD adults for (a) *mut-16(pk710)* alone or *mut-16(pk710)* animals carrying a *mut-16* rescue transgene expressed (b) pan-neuronally (*rab-3*p), c) in ADL neurons (*sre-1*p), d) in ASI neurons (*gpa-4*p), and (e) in ADF neurons (*srh-142*p). * *q* < 0.05, ** *q* < 0.01; Multiple unpaired *t*-tests with FDR correction. Additional data are provided in Supplementary Tables 2, 6–10.

We showed previously that MUT-16 expression was required in pheromone-sensing neurons to promote dauer formation in crowded conditions (Bharadwaj and Hall 2017). We reasoned that mating, a pheromone-dependent behavior, would also require MUT-16 function. To test the hypothesis that the RNAi pathways are required for increased outcrossing in postdauer populations, we performed mating assays using the *mut-16(pk710)* mutant strain. In the mating assay of *mut-16* control hermaphrodites and control males, we found an average mating efficiency lower than what was observed for wild type (0.095 ± 0.074) (Fig. 2a). Using a 2-way ANOVA, we found that the developmental history of the male was retained as a significant factor in mating efficiency in the *mut-16* strain (*P* = 0.018, 2-way ANOVA (Fig. 2a). We observed that the assays that included PD males had higher male frequencies compared to CON males when the hermaphrodite condition remained constant (0.205 ± 0.096 and 0.095 ± 0.074 for CON hermaphrodites, 0.166 ± 0.141 and 0.128 ± 0.099 for PD hermaphrodites). Indeed, when we compared mating efficiencies within a hermaphrodite type (either CON or PD) and the male type varied, we found significantly increased mating with PD males compared to CON males with CON hermaphrodites (*q* = 0.004; multiple unpaired *t*-tests). This difference in mating was not detectable within the PD hermaphrodite matings as observed in the wildtype strain (Fig. 2a). These results indicate that MUT-16 is required for the overall mating efficiency of N2 Bristol and the increased outcrossing phenotype in postdauer populations, likely through its function in PD hermaphrodites.

To further explore the neuronal role of MUT-16 in mating, we performed mating assays with strains that express a high-copy transgene array carrying the wild-type *mut-16* coding sequence either pan-neuronally or in individual, pheromone-sensing neurons. We showed previously that these transgenic strains rescued the dauer defective phenotypes exhibited by *mut-16* animals in high pheromone conditions (Bharadwaj and Hall 2017). To determine if MUT-16 was also functioning in these neurons to regulate mating behaviors, we performed the mating assay using a *mut-16* strain carrying a *mut-16* expression transgene driven by a pan-neuronal promoter (*rab-3*p). To our surprise, we found the mating efficiency in the *mut-16* pan-neuronal overexpression strain mating assays was suppressed well below baseline outcrossing and X-chromosome nondisjunction rates in *mut-16* mutants (0.005 ± 0.023 compared to 0.095 ± 0.074 for CON × CON matings) (Fig. 2b; see Materials and Methods). Despite the suppression of mating in the *mut-16* pan-neuronal rescue strain, we found that the developmental history of males continued to be a significant factor in outcrossing levels (*P* = 0.013, 2- way ANOVA). Inclusion of PD males in the mating assay increased the male frequency > 4 times compared to CON males, regardless of the hermaphrodite condition. We found that pan-neuronal rescue of MUT-16 was sufficient to restore the significant increase in outcrossing in postdauer populations compared to control mating assays observed in wildtype mating assays (*q* = 0.024, multiple unpaired *t*-tests) (Fig. 2b). Next, we attempted to perform mating assays using *mut-16* strains that carry overexpression transgene arrays driven by single-neuron promoters. We examined the mating efficiency of *mut-16* strains carrying the *mut-16* transgene expressed only in ADL neurons (*sre-1*p) and found that this strain also exhibited suppressed outcrossing below baseline observed for CON × CON in *mut-16* assays (0.025 ± 0.024 vs 0.095 ± 0.074) (Fig. 2c). In addition, mating assays using this strain does not show a significant source of variation from either sex, suggesting that overexpression of *mut-16* in ADL neurons is not sufficient to restore increased outcrossing in postdauer populations (Fig. 2c). We observed similar suppressed outcrossing levels and loss of variation due to male developmental history for the *mut-16* strains expressing the transgene in the pheromone-sensing ASI neurons (*gpa-4*p) (0.033 ± 0.029 vs 0.095 ± 0.074) (Fig. 2d). However, *mut-16* overexpression in ADF neurons (*srh-142*p) led to a similar mating suppression compared to *mut-16* as the other neuronal rescues tested here (0.033 ± 0.052 vs 0.095 ± 0.074), but also showed significant variation due to the life history of hermaphrodites (*P* = 0.023, 2-way ANOVA) (Fig. 2e). Mating assays including PD hermaphrodites exhibit higher male frequencies (0.131 ± 0.085 and 0.068 ± 0.088) compared to those with CON hermaphrodites (0.033 ± 0.052 and 0.013 ± 0.033). Only male ADF neurons are known to respond to ascr#3 to find mates (Fagan et al. 2018; Luo and Portman 2021; Luo et al. 2024); however, these results suggest that MUT-16 is required in PD hermaphrodites to promote outcrossing with males. Together, these results support the conclusion that *mut-16* expression is required pan-neuronally for promoting outcrossing in postdauer populations, although the individual neuron(s) required to express MUT-16 may not be the most characterized pheromone-sensing neurons tested here.

### Neuronal overexpression of *mut-16* negatively influences hermaphrodite fecundity

**Fig. 3.**
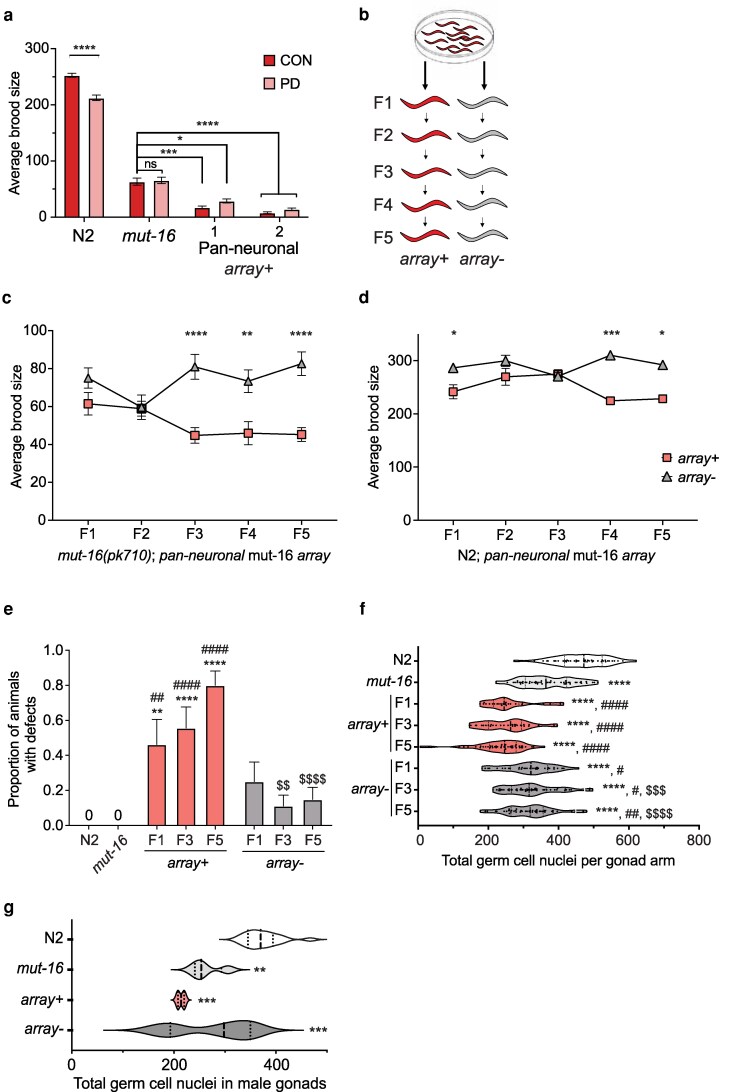
Neuronal overexpression of *mut-16* decreases hermaphrodite fecundity transgenerationally. a) Bar graph represents mean brood size ± S.E.M. for CON and PD hermaphrodites of wild type N2, *mut-16*(*pk710*), and *mut-16*(*pk710*) strains carrying the pan-neuronal *mut-16* expression array. * *P* < 0.05, *** *P* < 0.001, **** *P* < 0.0001, one-way ANOVA with Tukey's *post hoc* test. n.s. not significant. b) Diagram illustrating the experimental design of transgenerational brood size assays. c, d) Mean brood size ± S.E.M. for (c) *mut-16(pk710)* and (d) N2 strains carrying the pan-neuronal *mut-16* overexpression array (*array+*) or that have lost the array (*array−*) from F1 to F5 generations. * *P* < 0.05, ** *P* < 0.01, *** *P* < 0.001, **** *P* < 0.0001, multiple *t*-tests followed by Holm–Sidak correction. e) Mean proportion ± S.E.M. of hermaphrodite adults with small gonads in N2, *mut-16(pk710),* and *mut-16(pk710) array+* or *array−* strains. f, g) Violin plots represent the total number of germ cell nuclei for (f) hermaphrodite and (g) male gonad arms for N2, *mut-16(pk710),* and *mut-16(pk710) array+* or *array−* strains. e–g) *, #, $ *P* < 0.05; **, ##, $$ *P* < 0.01; ***, ###, $$$ *P* < 0.001; ****, ####, $$$$ *P* < 0.0001; one-way ANOVA with Tukey's *post hoc* test. * Denotes a comparison to N2; # denotes a comparison to *mut-16(pk710),* $ denotes a comparison to *array+* within the same generation. Additional data are provided in Supplementary Tables 11–13.

While performing the outcrossing assays with the *mut-16* pan-neuronal and individual neuron overexpression strains, we observed that they exhibited a decrease in total progeny and suppressed mating compared to wild-type and *mut-16* strains. To investigate whether suppressed outcrossing levels were due to germline defects, we first examined the ability of the *mut-16* neuronal overexpression strain hermaphrodites to produce self-fertilized progeny. We conducted brood size assays of CON and PD hermaphrodite adults with N2 Bristol, *mut-16*(*pk710*), and 2 independent lines of the *mut-16* strain carrying an extrachromosomal array of pan-neuronal *mut-16* expression transgene. First, we observed that wild-type postdauer hermaphrodites showed a significantly reduced brood size compared to controls, and that this difference is eliminated in the *mut-16(pk710)* strain, which is consistent with our previous reports (Fig. 3a) (Hall et al. 2010; Ow et al. 2018, 2021). Additionally, we found that control and postdauer adults of 2 independent pan-neuronal *mut-16* overexpression lines exhibited a significant reduction in brood size compared to both the wild-type and *mut-16* strains (Fig. 3a), indicating that the low fecundity of the overexpression strains was not merely due to the *pk710* allele alone. Thus, overexpression of *mut-16* in neurons has negative consequences on hermaphrodite fecundity in *mut-16* mutants.

### The negative effects of MUT-16 overexpression on hermaphrodite fecundity are transgenerationally inherited

Double-stranded RNA (dsRNA), the trigger for RNAi, has been shown to regulate germline gene expression transgenerationally when expressed in neurons (Devanapally et al. 2015; Posner et al. 2019; Ewe et al. 2025). Thus, we questioned whether the reduced brood size observed in the *mut-16* pan-neuronal overexpression strains could be inherited over generations that have lost the *mut-16* expression transgene. To address this question, we counted the brood sizes of F1 to F5 generations of *mut-16* hermaphrodites that have retained (*array+*) or lost (*array−*) the extrachromosomal *mut-16* transgene array in the F1 generation from an *array+* hermaphrodite parent (see Materials and Methods) (Fig. 3b). The F1 and F2 generations did not exhibit a significant difference in brood size between *array+* and *array−* populations. However, for the F3 through F5 generations, we observed that the *array−* populations had significantly larger brood sizes compared to their *array+* counterparts (Fig. 3c). This result was due to a gradual increase and decrease in fecundity of the *array−* and *array+* progeny, respectively, over the 5 generations examined. In *mut-16* animals, transposon activity is derepressed, resulting in increased mutagenesis and decreased genome integrity (Ketting et al. 1999; Tabara et al. 1999; Sijen and Plasterk 2003). We therefore asked if the decline in hermaphrodite fecundity of the *mut-16* pan-neuronal overexpression strains was due to accumulated mutations in the sensitized *mut-16*(*pk710*) background and hypothesized that the neuronal rescue would not be sufficient to rescue the transposon suppression defects in other tissues, including the germline. To address this question, we injected the wild-type N2 strain with the pan-neuronal *mut-16* expression transgene array and examined the brood sizes of *array+* and *array−* progeny of the F1 to F5 generations from an *array+* hermaphrodite. For the F1 through F3 generations, the *array+* populations show variable, but similar, brood sizes to the *array−* progeny (Fig. 3d). In the F4 and F5 generations, we observed a similar difference in brood sizes compared to the *mut-16(pk710)* strain, where brood sizes of *array+* adults were significantly smaller than *array−* adults (Fig. 3d). Together, these results suggest that the neuronal overexpression of *mut-16* is correlated with increased sterility over generations, possibly through inheritance of epigenetic factors such as small RNAs.

### Neuronal overexpression of *mut-16* negatively influences gonad morphology and germ cell number transgenerationally

In previous work by others, *mut-16* mutants were shown to exhibit germline defects following heat stress, including small or collapsed gonad arms, defects in maintaining germ cell pluripotency, and misregulation of spermatogenic, oogenic, and somatic genes in the germline (Rogers and Phillips 2020a, 2020b). Thus, we asked whether neuronal overexpression of *mut-16* worsened the gonad morphology defects of *mut-16* animals, leading to sterility. Since the decreased fecundity is more severe in animals retaining the array compared to *mut-16(pk710)* animals (Fig. 3a), we expected to observe a correlation of gonad morphology defects with *array+* animals that were not present in the *array−* counterparts. To test this hypothesis, we examined the somatic gonad and germline morphology for defects in the F1, F3, and F5 generations of *mut-16* pan-neuronal overexpression strain hermaphrodites that either retained (*array+*) or lost (*array−*) the extrachromosomal array. As reported previously, the proportion of *mut-16(pk710)* animals displaying overall gonad defects was significantly higher than that of N2 Bristol, including DTC migration defects, decreased sperm number, and thin distal gonads (Supplementary Figs. 4 and 5). However, for small gonad defects, we detected a trend in the proportion of animals exhibiting this defect and the observed brood sizes of these populations. Interestingly, the *array+* populations showed an increasing proportion of defects between the F1 and F5 generations, while the *array−* populations exhibited significantly fewer defects (Fig. 3e). Moreover, neither wild-type N2 nor *mut-16* strains exhibited the small gonad phenotype, suggesting that the defect was a consequence of the pan-neuronal *mut-16* array.

To further characterize how the neuronal overexpression of *mut-16* contributes to decreased outcrossing, we examined whether small gonad defects were correlated with fewer germ cells available for reproduction. Using DAPI-stained whole hermaphrodites, we quantified the number of germ cells per gonad arm of *array+* and *array−* progeny for F1, F3, and F5 generations and compared it to wild-type and *mut-16(pk710)* strains. As expected, we found that *mut-16(pk710)* hermaphrodites exhibited a significant decrease in germ cell counts compared to wild type (Fig. 3f). Furthermore, we observed that *array+* generations exhibited a further decrease in total germ cells compared to *mut-16* adults, and the *array−* animals exhibited an intermediate germ cell number between the *mut-16* and *array+* populations (Fig. 3f). Upon further scrutiny, we found that the differences in total germ cell number appear to be due to a decrease in cells in the mitotic and pachytene regions of the germline (Supplementary Figs. 6 and 7). This observation is consistent with germline *Mutator* foci having the highest expression in the mitotic and transition zones and the late pachytene region (Uebel et al. 2018). We next examined whether the pan-neuronal *mut-16* array resulted in similar effects in male germlines. We found that wild-type males contain an average of 373.6 ± 7.9 germ cells in their gonads (Fig. 3g). Male *mut-16* adults exhibit a significant decrease in germ cell numbers (262.5 ± 15.0), which is not significantly exacerbated by expression of the *mut-16* pan-neuronal array (Fig. 3g). The differences in germ cell number between wild-type and *mut-16* strains are due to significantly fewer mitotic germ cells in male gonads (Supplementary Fig. 7d). Together, these results indicate that the overexpression of MUT-16 in neurons can negatively impact hermaphrodite fecundity and germ cell numbers transgenerationally, while not significantly affecting male germ cell numbers beyond the *mut-16* baseline defects. Due to the heritable, negative effects of *mut-16* overexpression on fecundity and germline morphology in hermaphrodites, we were unable to further investigate the role of MUT-16 in outcrossing levels using these strains.

### TGF-β signaling pathway is required for increased outcrossing in postdauer populations

**Fig. 4.**
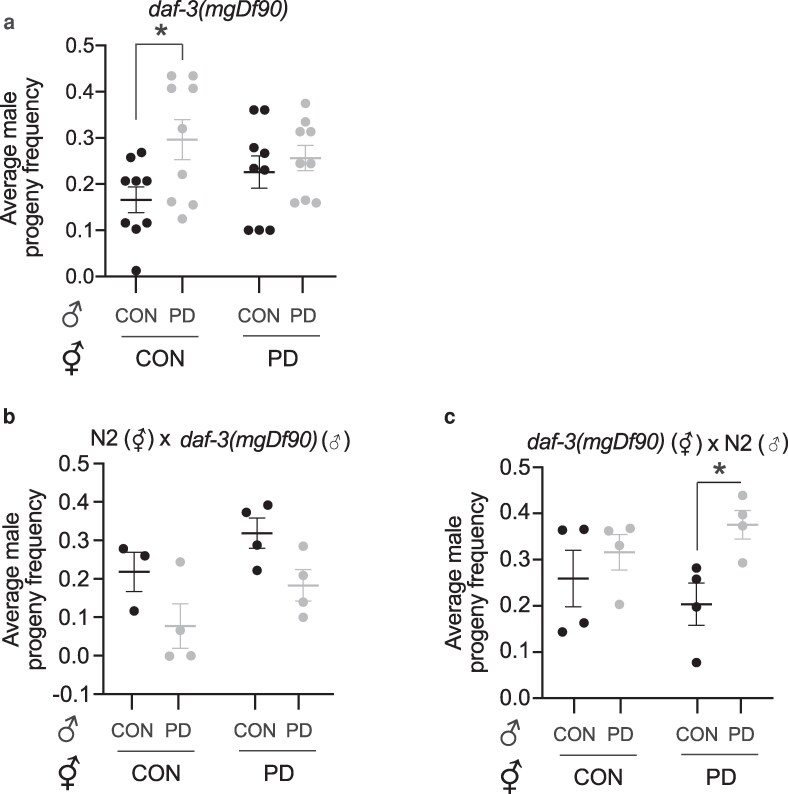
TGF-β signaling plays a role in increased outcrossing levels in postdauer populations. Scatterplots with lines representing the average male progeny frequencies ± S.E.M. for mating assays including CON and PD populations of (a) *daf-3*(*mgDf90*), b) N2 hermaphrodites mated with *daf-3* males, and (c) *daf-3* hermaphrodites mated with N2 males. * *q* < 0.05, Multiple unpaired *t*-tests with FDR correction. Additional data provided in Supplementary Tables 2, 14–16.

We previously showed that the *osm-9* TRPV channel gene is regulated by the TGF-β pathway in ADL neurons of postdauer hermaphrodites. Specifically, the DAF-3/co-SMAD transcription factor binds to the *osm-9* promoter to promote its downregulation after dauer, and mutations in *daf-3* result in continued *osm-9* expression and ascr#3 avoidance in postdauer hermaphrodites (Sims et al. 2016). Since we have shown that *osm-9* is required for the increased mating efficiency in postdauer populations, we tested whether the increased outcrossing levels in postdauer populations were dependent upon TGF-β signaling by performing mating assays using a *daf-3(mgDf90)* mutant strain. For mating assays of *daf-3* CON hermaphrodites and CON males, we observed a similar baseline of mating efficiency to what we observed for wild type (0.166 ± 0.028) (Figs. 4a). In addition, we continue to observe that the life history of the male is a significant factor of variation (*P* = 0.025, 2-way ANOVA). However, when we tested whether the male frequencies were significantly different when CON or PD males were included with the hermaphrodite condition remaining constant, we detected a significant increase in mating efficiency in assays with PD males compared to CON males in mating assays with CON hermaphrodites (*q* = 0.023, multiple unpaired *t*-tests). Interestingly, the male frequency levels for the *daf-3* mating assays look strikingly similar to the *mut-16* assays (Figs. 2a and 4a). These results indicate that DAF-3 is required to promote outcrossing in PD populations.

To further explore this result, we next asked whether DAF-3 was required in males or hermaphrodites to promote postdauer outcrossing. Given that *daf-3* mutants continue to show increased mating efficiencies with postdauer males, we predicted that the expression of *daf-3* is required in hermaphrodites to significantly increase outcrossing in postdauer populations. To test our hypothesis, we examined mating efficiency where only one sex carrying a mutant allele of *daf-3* was mated with wild-type animals of the other sex. In populations where *daf-3* males were mated to wild-type hermaphrodites, we continue to observe that the male life history is a significant contributor to variation, similarly to *daf-3* mating assays (*P* = 0.012, 2-way ANOVA). However, in this case, we found that the trends in outcrossing were the opposite compared to wild type (Fig. 4b). CON males mated to CON hermaphrodites exhibited a mating efficiency of 0.216 ± 0.099, higher than that observed in assays that were exclusively wild-type or *daf-3* adults. Mating assays that included PD males showed a trend of decreased mating efficiency compared to mating assays that included CON males (0.075 ± 0.109 and 0.175 ± 0.077 vs 0.216 ± 0.099 and 0.322 ± 0.085; Fig. 4b). These results are consistent with a requirement for DAF-3 in postdauer males to promote outcrossing, although the genotypic context (wildtype vs *daf-3* hermaphrodites) in which males are DAF-3-deficient impacts mating efficiency. The genotype of the hermaphrodite is also critical as the outcrossing of *daf-3* mutant populations show a distinct trend compared to the populations where only males are *daf-3* deficient.

We further investigated a potential role of DAF-3 in outcrossing by examining the reciprocal cross of *daf-3* hermaphrodites with wild-type males. If hermaphrodites did not play a significant role in outcrossing levels, we would expect that mating efficiencies of these assays would be similar to wild type. Similar to the previous mating assays including *daf-3* mutants, the life history of the male was a significant factor in the variation of mating efficiency (*P* = 0.020, 2-way ANOVA). When we tested whether outcrossing in assays with either CON or PD males for a hermaphrodite type were significantly different, we found that crosses with PD males had an increased mating efficiency compared to CON males for the assays with PD hermaphrodites (*q* = 0.019, Multiple unpaired *t*-tests; Fig. 4c). This result indicates that DAF-3 is required in postdauer males to promote increased outcrossing. Furthermore, the observation that restoration of DAF-3 in males does not completely recapitulate the increased outcrossing in postdauer populations compared to control populations further suggests a minor role of DAF-3 in hermaphrodites (Figs. 1a and 4c). Together, these results indicate that DAF-3 is required in both males and hermaphrodites to promote outcrossing in postdauer populations.

### Postdauer males exhibit increased ability to detect mates via pheromone

Males find mates through detection and chemotaxis toward pheromone molecules produced by hermaphrodites (Srinivasan et al. 2008; Leighton et al. 2014; Fagan et al. 2018; Luo et al. 2024). Our results indicate that the developmental history of the male is a significant contributor to outcrossing rates, with hermaphrodites playing a more minor role. We hypothesized 2 nonmutually exclusive mechanisms to explain how postdauer males contribute to outcrossing levels. First, postdauer males may be more efficient at finding mates than control males. A second possibility is that postdauer males may produce a novel pheromone component that functions as an attractant to hermaphrodites.

**Fig. 5.**
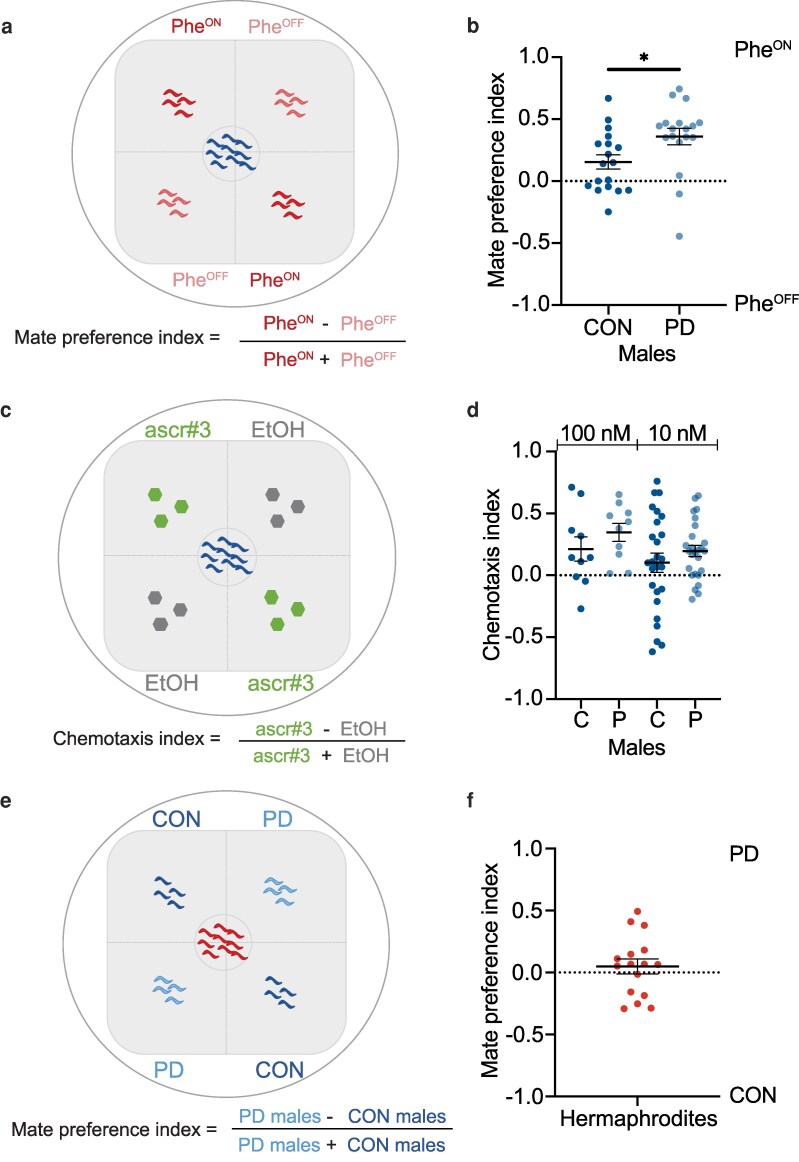
Postdauer males exhibit an increased ability to locate mates via pheromone. a, e) Schematic for the mate preference and (c) chemotaxis assay experimental setups. a) Immobilized hermaphrodites of the same genotype (Phe^ON^: *unc-54* or Phe^OFF^: *unc-54; daf-22*) were placed on opposite quadrants and, b) males were scored based on quadrant location. c, d) CON and PD *him-5* male preference for ascr#3 or EtOH quadrants. e) Immobilized CON and PD *him-5* males were placed in opposite quadrants, and (f) hermaphrodites were scored based on quadrant location. *N* ≥ 10 trials; *n* ≥ 100 animals. * *P* < 0.05, unpaired *t*-test. Additional data are provided in Supplementary Table 17.

To test these possibilities, we first performed mate preference assays using genetically immobilized hermaphrodites to determine male preference and their ability to locate mates (Fagan et al. 2018; Luo et al. 2024). Each quadrant contained immobilized control hermaphrodites that can (Phe^ON^: *unc-54*) or cannot (Phe^OFF^: *unc-54; daf-22*) secrete ascaroside pheromones into their environment. In each assay, either control or postdauer wild-type males were placed in the center of the plate and scored for their location in the quadrants after 30, 60, and 90 min (Fig. 5a). Both control and postdauer males exhibited preference toward hermaphrodites that were able to secrete pheromones (Fig. 5b). Interestingly, we observed that postdauer males were better able to locate the pheromone-producing hermaphrodites compared to control males (Fig. 5b). These results support the hypothesis that postdauer males are more efficient at locating mates compared to control males. To test if the increased postdauer male efficiency at locating mates was facilitated by ascr#3 detection, we also conducted chemotaxis quadrant assays with ascr#3 at 10 nM, a concentration that does not elicit dwelling, and 100 nM, a concentration that elicits dwelling in males (Narayan et al. 2016; Fagan et al. 2018; Luo et al. 2024). Males were tested for their preference to 4 quadrants containing either ascr#3 or ethanol (Fig. 5c). Postdauer males exhibited a slight, but statistically insignificant, increase in chemotaxis toward both 10 and 100 nM ascr#3 concentrations compared to control males (Fig. 5d). This result was not surprising given that males are attracted to a blend of pheromones secreted by hermaphrodites, and ascr#3 detection may be just one contributor to the increased mate finding efficiency of postdauer males.

To test the possibility that postdauer males secrete a pheromone for hermaphrodite attraction, we again performed the mate preference quadrant assays using immobilized (*unc-54*) control or postdauer males in each quadrant. The location of control wild-type hermaphrodites was scored every 30 min, up to 90 min (Fig. 5e). In contrast to the assay for male preference, hermaphrodites did not exhibit any preference between control and postdauer males (Fig. 5f). Together, these results suggest that postdauer males promote outcrossing through their increased ability to locate potential mates using pheromone cues.

## Discussion

Phenotypic plasticity plays a significant role in regulating reproductive modes. Here, we show that the change in reproductive strategies to increase mating in *C. elegans* following dauer diapause requires sexually dimorphic changes in behavior. First, the developmental history of males was the major contributor to outcrossing levels, partially due to the increased ability of postdauer males to detect mates using pheromone cues compared to control males. In addition, we uncovered a minor role for the developmental history of hermaphrodites in regulating mating levels. These phenotypes depend on RNAi and TGF-β pathways, which potentially act to regulate pheromone detection. Furthermore, we demonstrate that overexpression of MUT-16 pan-neuronally or in individual neurons negatively regulates hermaphrodite fecundity by decreasing germ cell number transgenerationally. Together, our results highlight a subset of the genetic determinants required for increased outcrossing behaviors in postdauer populations.

### Contributions of males and hermaphrodites toward outcrossing efficiency

The original hypothesis driving this work was that downregulation of *osm-9* expression in postdauer hermaphrodite ADL neurons facilitated the opportunity for increased mating due to their failure to avoid high concentrations of ascr#3. However, our results indicated that the developmental history of males was the primary determinant of outcrossing rates, with hermaphrodite life history playing a smaller role (Fig. 1a). We showed that OSM-9 is required for the increase in mating efficiency, but expression in ADL alone is not sufficient for increased outcrossing levels in postdauer populations (Fig. 1b and Supplementary Fig. 2a). *C. elegans* males balance their drive for reproduction and survival by alternating between foraging (food-seeking) and exploration (mate-seeking) behaviors. Male exploration is suppressed by the presence of hermaphrodites and governed by the nutritional status of the males (Lipton et al. 2004; Barrios et al. 2008, 2012; Ryan et al. 2014; Wexler et al. 2020; Luo and Portman 2021; Luo et al. 2024). Exploratory behavior is regulated by the ray neurons and AWA and ASJ sensory neurons (Barrios et al. 2008; Ryan et al. 2014; Hilbert and Kim 2017, 2018; Wexler et al. 2020); a change in the activity of these neurons in postdauer males could modulate the switch between foraging and exploration behaviors more efficiently or more frequently than control males, resulting in the increased outcrossing rate we observed (Gray et al. 2005). This hypothesis is supported by other reports showing that *C. elegans* hermaphrodites of the Hawaiian wild isolate CB4856 can permanently alter their foraging behavior based on passage through the starvation-induced dauer stage (Pradhan et al. 2019). While this change in foraging behavior is not seen in N2 Bristol postdauer hermaphrodites, the possibility exists that males of this strain experience a sex-specific change in their foraging behavior in response to starvation-induced dauer.

Hermaphrodites influence males to switch from foraging behavior to mate-locating behavior by secreting a mixture of “mate-finding” pheromone molecules (Simon and Sternberg 2002; Lipton et al. 2004). Males are attracted to a particular blend of ascarosides that includes ascr#2, ascr#3, ascr#4, and ascr#8, with males being most attracted to ascr#3 (Srinivasan et al. 2008; Kaplan et al. 2011; Narayan et al. 2016). In this study, we showed that postdauer males exhibited similar acute ascr#3 avoidance as control males (Fig. 1d). However, in a quadrant assay, postdauer males exhibited a trend of increased attraction, albeit nonsignificant, toward ascr#3 compared to control males (Fig. 5d). In quadrant assays, male ascr#3 attraction is mediated by the ADF sensory neurons and the PDFR-1-dependent inhibition of a nonsex-specific avoidance behavior promoted by ASI (Fagan et al. 2018; Luo and Portman 2021; Luo et al. 2024). Plasticity in the activity or connections of these neurons, perhaps through altered *osm-9* expression, may contribute to the increased ability of postdauer males to locate mates. Indeed, cilia, amphid sheath cells, and electrical synapses have all been shown to remodel in dauers to contribute to dauer-specific behaviors (Albert and Riddle 1983; Procko et al. 2011; Schroeder et al. 2013; Bhattacharya et al. 2019). Interestingly, the fusion of amphid sheath cells in dauers, which interact with the ASI, AWC, ADF, ASK, and ADL amphid sensory neurons, is irreversible and may contribute to behavioral plasticity of chemotaxis behaviors in postdauer males (Procko et al. 2011).

One of the minor roles of postdauer hermaphrodites contributing to increased outcrossing was uncovered through reciprocal crosses with wild type and *daf-3*/co-SMAD mutants (Fig. 4). In wild-type populations, the mating level of postdauer hermaphrodites and males was significantly greater than control hermaphrodites and males, but that difference was lost in crosses that included *daf-3* hermaphrodites (Figs. 1a and 4). In addition, the observation that *daf-3* postdauer males correlated with decreased mating in the presence of wild type hermaphrodites, but not *daf-3* hermaphrodites, suggests that both the developmental history and genetic background of each sex may interact to regulate mating levels. However, the role of *daf-3* in regulating outcrossing sex-specifically is not clear. One possible explanation is that *daf-3* mutants exhibit significantly increased path range, as well as changes in foraging and crawling frequency, compared to wild-type adults (Yemini et al. 2013). These changes in the relative time spent in exploration vs foraging modes could alter mating efficiencies in crosses containing *daf-3* mutants compared to wild type. Another potential explanation is that TGF-β signaling may alter ascaroside biosynthesis, which is regulated by diet and metabolic state (Kaplan et al. 2011). Postdauer hermaphrodites secrete a greater amount of ascr#4, whereas control hermaphrodites predominantly secrete ascr#2 (Kaplan et al. 2011). Interestingly, males linger in regions containing ascr#3 and ascr#4 longer than regions containing ascr#3 and ascr#2, suggesting that postdauer hermaphrodites may be more attractive to males compared to controls (Srinivasan et al. 2008). Although a direct link between *daf-3* and ascaroside biosynthesis has not been established to date, TGF-β signaling has been shown to regulate diverse processes such as dafachronic acid biosynthesis, dauer formation, sperm motility, germline proliferation, and lipid metabolism based on environmental conditions (Thomas et al. 1993; Gerisch and Antebi 2004; McKnight et al. 2014; Hussey et al. 2017; Pekar et al. 2017).

### Transgenerational regulation of germ cell number by neuronal RNAi

In previous work, we demonstrated that *mut-16* hermaphrodites exhibited a dauer deficiency phenotype in high pheromone conditions due to their decreased expression of G proteins which are required in chemosensory neurons to respond to environmental cues (Bharadwaj and Hall 2017). Although only hermaphrodites were examined previously, it is possible that MUT-16 is also required in males in order to appropriately respond to ascaroside molecules. Given that mating occurs through a pheromone-based mechanism, we predict that *mut-16* males are unable to mate with hermaphrodites due to their inability to detect mates via ascarosides. We also showed that *mut-16* males possess fewer mitotic germ cells in their gonads, which is consistent with *Mutator* foci being expressed in male germlines (Fig. 3g and Supplementary Fig. 7d). MUT-16 is also expressed in spermatid cytoplasm, and *mut-16* male sperm may also have defects not examined here that contribute to the decreased mating efficiency in this strain (Uebel et al. 2020). While overexpressing *mut-16* neuronally rescued the larval dauer deficiency, it failed to restore wild-type levels of male frequencies in mating assays and further reduced the mating efficiency compared to *mut-16* mutants without the transgene array (Fig. 2) (Bharadwaj and Hall 2017). We hypothesize that the failure of the transgene array to rescue the overall outcrossing levels in *mut-16* animals was due to its effects in the germline rather than in neurons, since expression of the pan-neuronal *mut-16* array resulted in male progeny frequency trends similar to wild type (Fig. 2b). MUT-16 is expressed in somatic and germline tissues and promotes formation of phase-separated condensates, called *Mutator* complexes, in the germline (Phillips et al. 2012; Uebel et al. 2018). *Mutator* complexes are localized perinuclearly along with P granules, which function together to amplify siRNAs (Zhang et al. 2011; Phillips et al. 2012; Uebel et al. 2018). Interestingly, overexpression of MUT-16 in muscle has been shown to drive formation of *Mutator* complexes in those cells, suggesting the possibility that our neuronal overexpression of *mut-16* may have similar consequences (Uebel et al. 2018). However, how the function of neuronal MUT-16 changes when diffuse in the cytoplasm compared to localized in *Mutator* complexes remains unclear.

A second question raised by this work is how MUT-16 function in the neurons is regulating gonad development and germ cell number. Mutant *mut-16* hermaphrodites have brood sizes that are approximately half of what is observed for wild type, and the strains overexpressing *mut-16* in neurons exhibit even further reduced broods and additional defects in gonad size and germ cell number (Figs. 3, Supplementary Figs. 4–7) (Rogers and Phillips 2020a, 2020b). Double-stranded RNA expressed in neurons has been shown to regulate gene expression in the germline transgenerationally (Devanapally et al. 2015). Given the role of MUT-16 in siRNA amplification, one likely explanation of our results is that overexpression of *mut-16* leads to accumulation of siRNAs in neurons that are transported to the germline where they inappropriately alter gene expression, resulting in increased sterility (Figs. 3, Supplementary Figs. 4–7). Future work could investigate the mechanism of siRNA travel between tissues and what germline genes are being misregulated.

Contextualizing these findings in evolutionary theory is important, as these changes in reproductive strategies may be contributing to adaptation. Changes in outcrossing rates and differential gene expression patterns are all likely to be mechanisms promoting populations to adapt to their environment by increasing allelic diversity. Here, we show a genetic mechanism underlying the switch of reproductive systems from primarily self-fertilization to a mixed mating system in response to environmental stress during early development. Males are more efficient at entering dauer diapause, have increased survival while in the dauer stage, are actively attracted to dauer-inducing pheromones, and are better able to locate mates following dauer (Ailion and Thomas 2000; Morran et al. 2009; Ludewig and Schroeder 2013). These characteristics make males a tool for driving genetic diversity after dauer exposure. Since natural populations of *C. elegans* are often found in dauer, these mechanisms to increase genetic diversity in postdauer populations are likely to have profound effects on their genome evolution as well as the ability to adapt to future stressors (Frézal and Félix 2015).

## Supplementary Material

jkag146_Supplementary_Data

## Data Availability

Raw data and Supplemental figures for this study are included as Supplemental files associated with this manuscript and can be accessed through GSA online. Animal strains and other reagents will be provided upon request after publication. Supplemental material available at G3 online.
