# Supplementary material for: Mating strategies in Caenorhabditis elegans populations are determined by male developmental history
Source: G3 (Bethesda). 2026 Jun 23;16(8):jkag146. doi: 10.1093/g3journal/jkag146 (PMC13439940; doi:10.1093/g3journal/jkag146)
Supplement: Supplementary file 1 [file jkag146_supplementary_data.zip › Supplemental_Figures_G3-2026-406924.pdf]

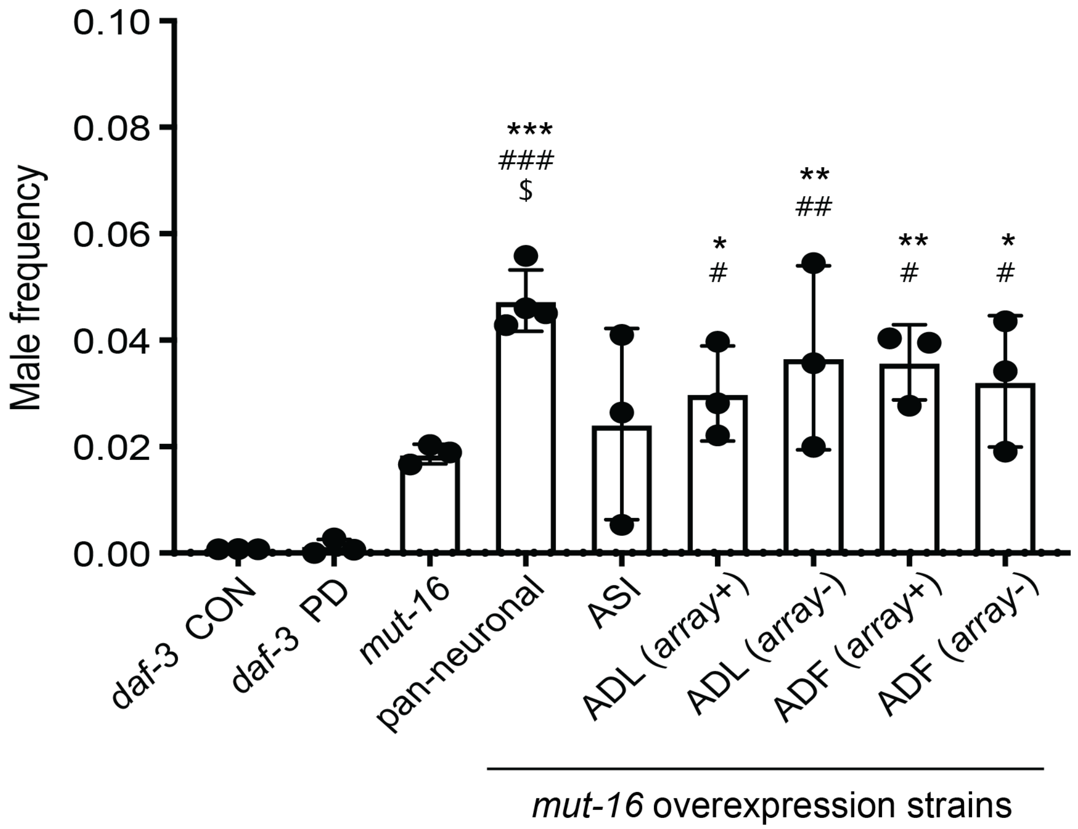

Figure S1. *mut-16* neuronal overexpression strains exhibit increased male incidence. Graph represents the mean male frequency ± S.E.M. n ≥ 18 animals over at least 3 biologically independent trials. The value for N2 male frequency is ≤ 0.002 as reported by Teotónio *et al.* (2006). *, #, $ indicate $p < 0.05$; **, ## indicate $p < 0.01$; ***, ### indicate $p < 0.001$. * denotes a comparison to *daf-3* CON; # denotes a comparison *daf-3* PD; $ denotes a comparison to *mut-16*; one-way ANOVA with Tukey's *post hoc* test. Additional data are provided in Table S3.

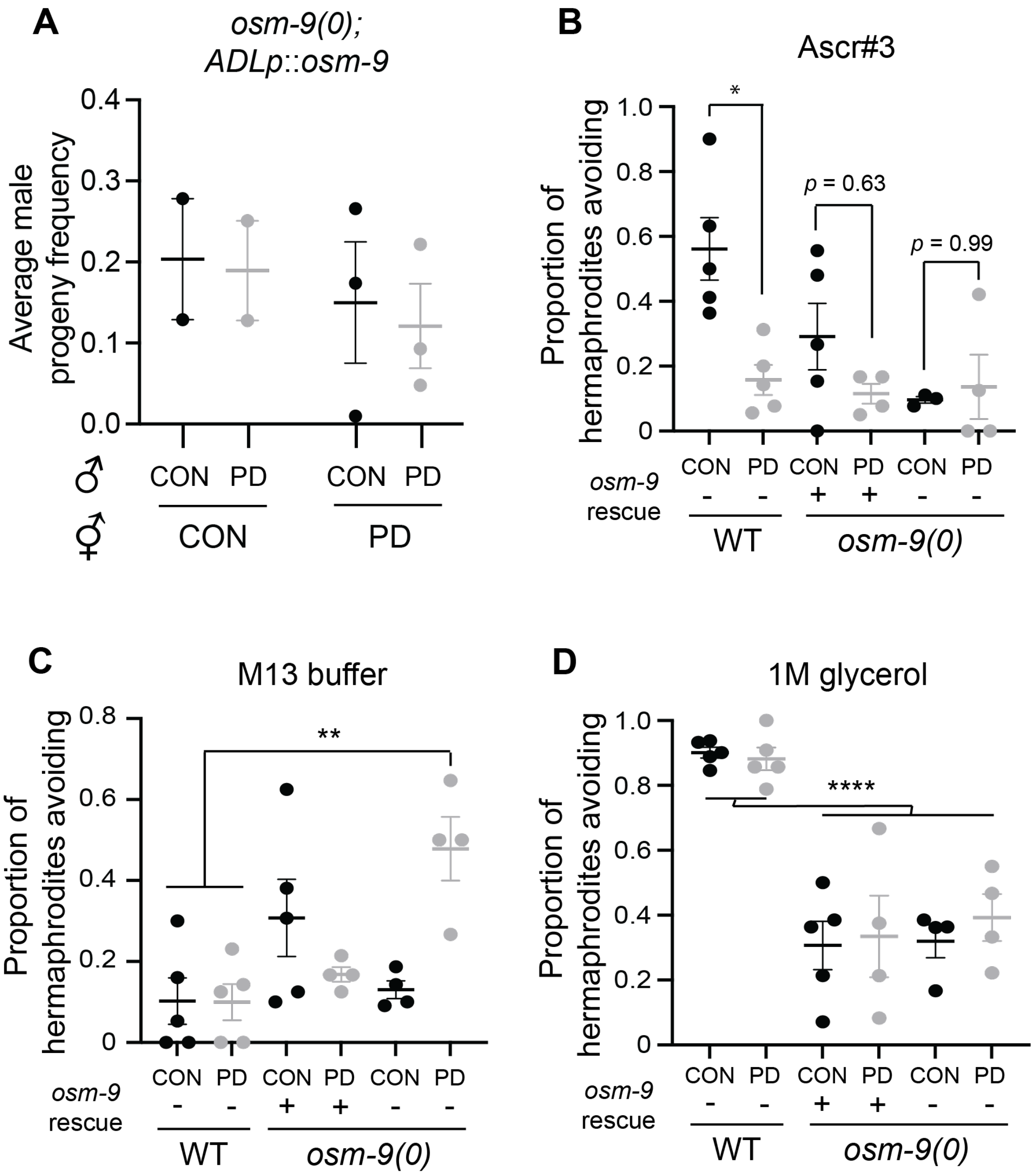

Figure S2. OSM-9 in ADL is not sufficient for increased outcrossing phenotype in postdauer adults. (A) Scatter plots with lines representing mean ± S.E.M. male progeny frequencies resulting from mating assays including combinations of CON and PD adults for *osm-9(0); ADL*p::*osm-9* rescue strain. Additional data are provided in Tables S2 and S4. **(B, C, D)** Mean avoidance ± S.E.M. for hermaphrodites and males in WT, *osm-9(0)*, and *osm-9(0); ADL*p*::osm-9* rescue strains in response to (B) ascr#3, (C) M13 buffer, and (D) 1M glycerol. * $p < 0.05$, ** $p < 0.01$, **** $p < 0.0001$; one-way ANOVA with Tukey's multiple comparisons test. Additional data are provided in Table S5.

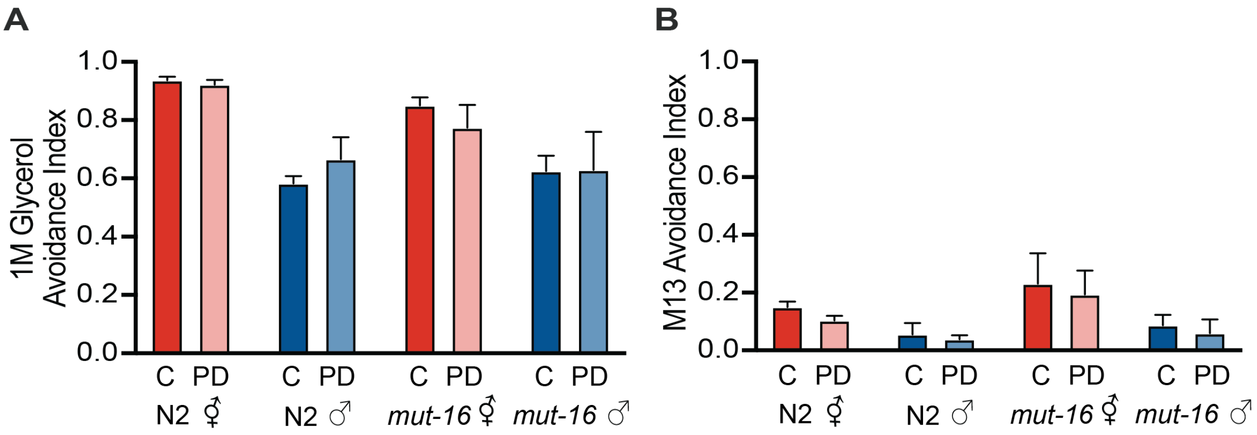

Figure S3. 1M glycerol and M13 avoidance indices for N2 and *mut-16* strains. Graphs represent mean avoidance index ± S.E.M. for wild type N2 and *mut-16(pk710)* hermaphrodites and males in response to **(A)** the positive control, 1M glycerol, and **(B)** the negative control, M13 buffer. n ≥ 76 animals over at least 3 biologically independent trials. CON and PD data within a strain are not significantly different using multiple *t*-tests. Additional data is provided in Table S5.

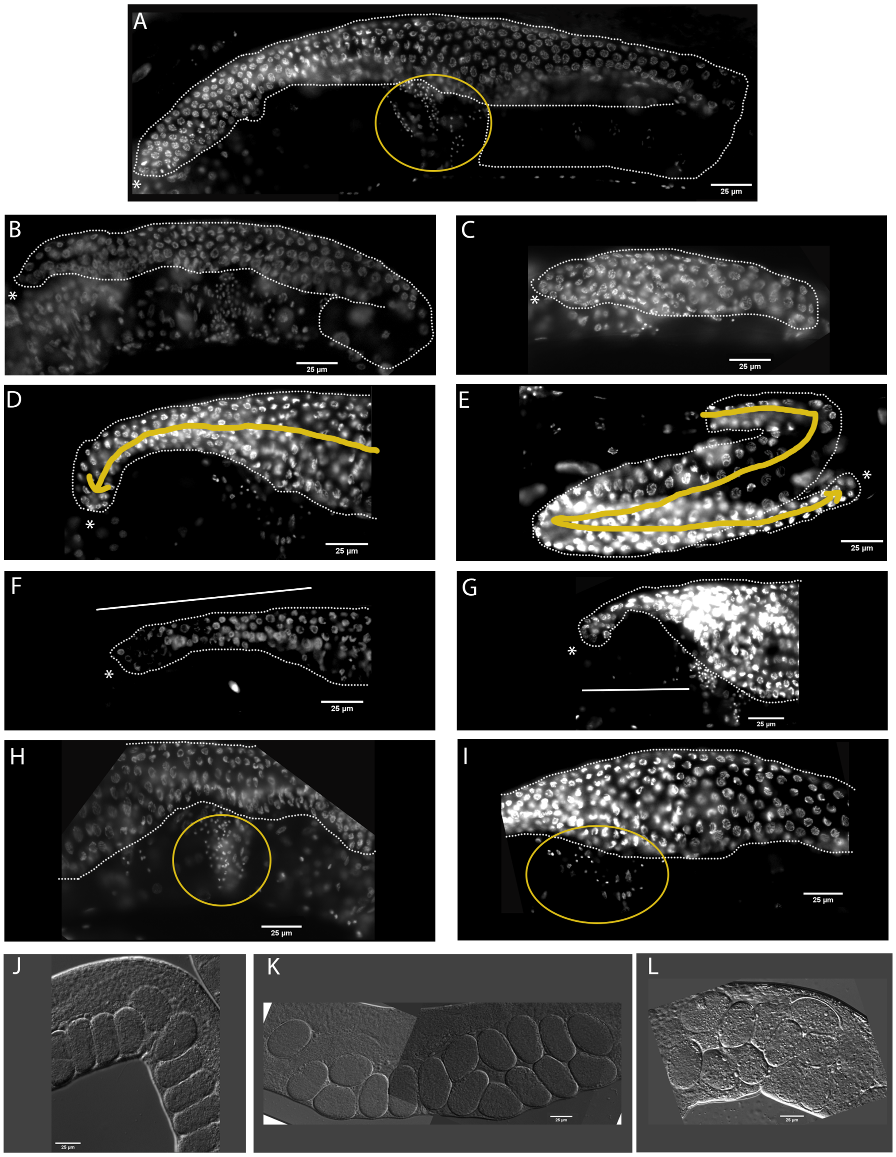

Figure S4. Presence of *mut-16* overexpression transgene in neurons correlates with numerous gonadal defects in *mut-16(pk710)* hermaphrodites. (A) Image is a representative wild-type N2 gonad. **(B, C)** Images represent **(B)** mild and **(C)** severe examples of a small gonad. **(D, E)** Images represent **(D)** mild and **(E)** severe examples of DTC migration defects. The yellow arrow indicates the probable path the DTC has taken. **(F, G)** Images represent examples of a **(F)** thin and an **(G)** extremely thin distal gonad size. The line indicates the gonad areas exhibiting the phenotype. **(H, I)** Images represent examples of **(H)** low to **(I)** extremely low sperm number. The yellow circle indicates the spermatheca. **(J)** Image represents an example of a wild-type N2 uterus. **(K, L)** Images represent examples of **(K)** mild and **(L)** severe embryo retention in *mut-16* overexpression strains. For all images, white dotted lines demark the gonad and the asterisks indicate the DTC. Scale bar is 25µm. **(A-I)** Images are oriented with the DTC towards the left side, except for **(E)** where the gonad has an additional turn.

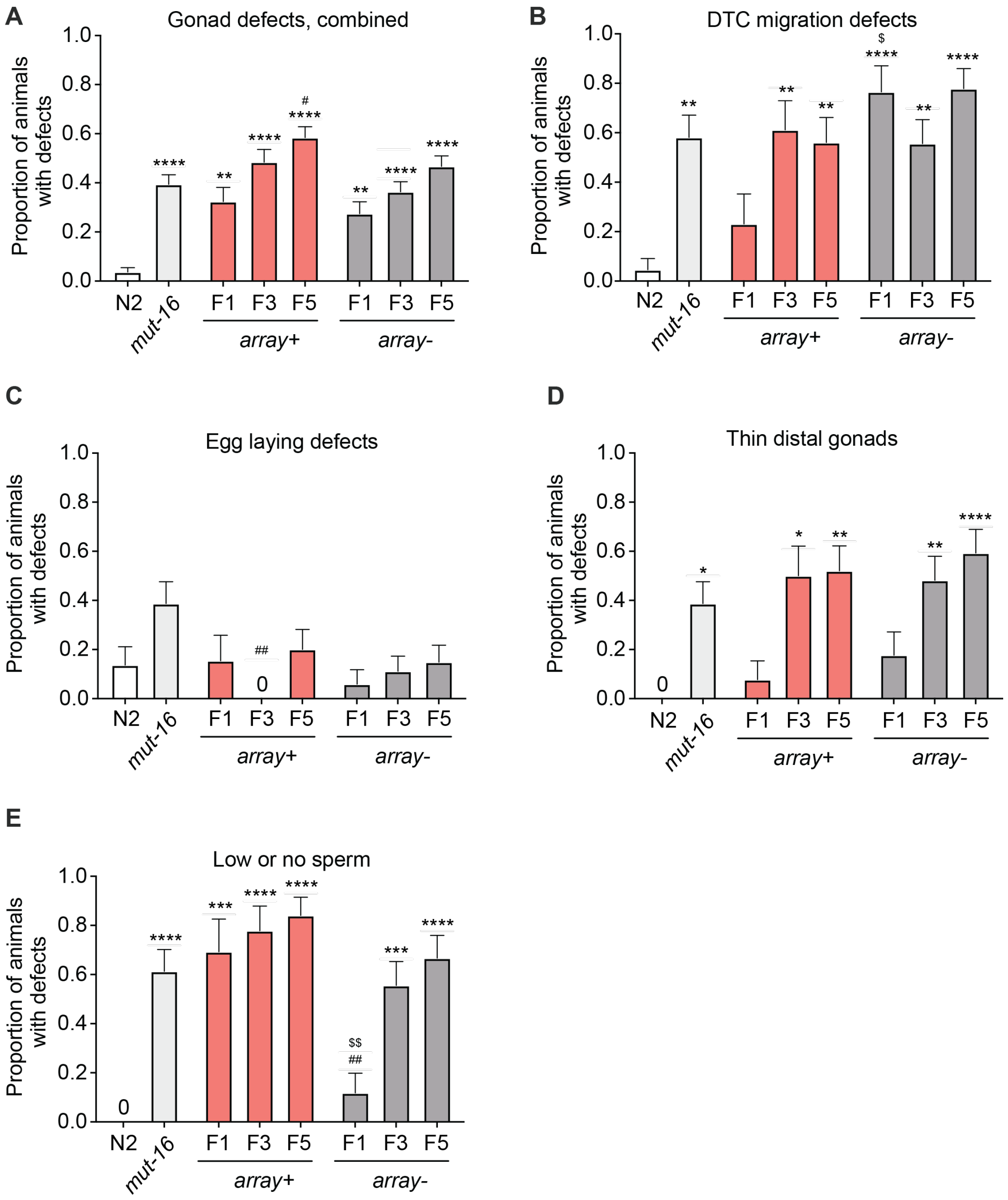

Figure S5. *mut-16* hermaphrodites exhibit numerous gonadal defects. The bar graphs represent mean proportion of animals with germline defects ± S.E.M. in wild type N2, *mut-16(pk710),* and F1, F3, and F5 generations of *mut-16(pk710) array*+ and *array-* strains. The germline defects examined were **(A)** total gonadal defects, **(B)** DTC migration defects, **(C)** egg laying defects, **(D)** thin distal gonads, and **(E)** low or no sperm. n ≥ 25 gonad arms over 3 biologically independent trials. *, #, $ $p < 0.05$; **, ##, $$ $p < 0.01$; ***, ###, $$$ $p < 0.001$; ****, ####, $$$$ $p < 0.0001$; one-way ANOVA with Tukey's *post hoc* test. * denotes a comparison to N2; # denotes a comparison to *mut-16(pk710),* $ denotes a comparison to *array*+ within the same generation. Additional data are provided in Table S13.

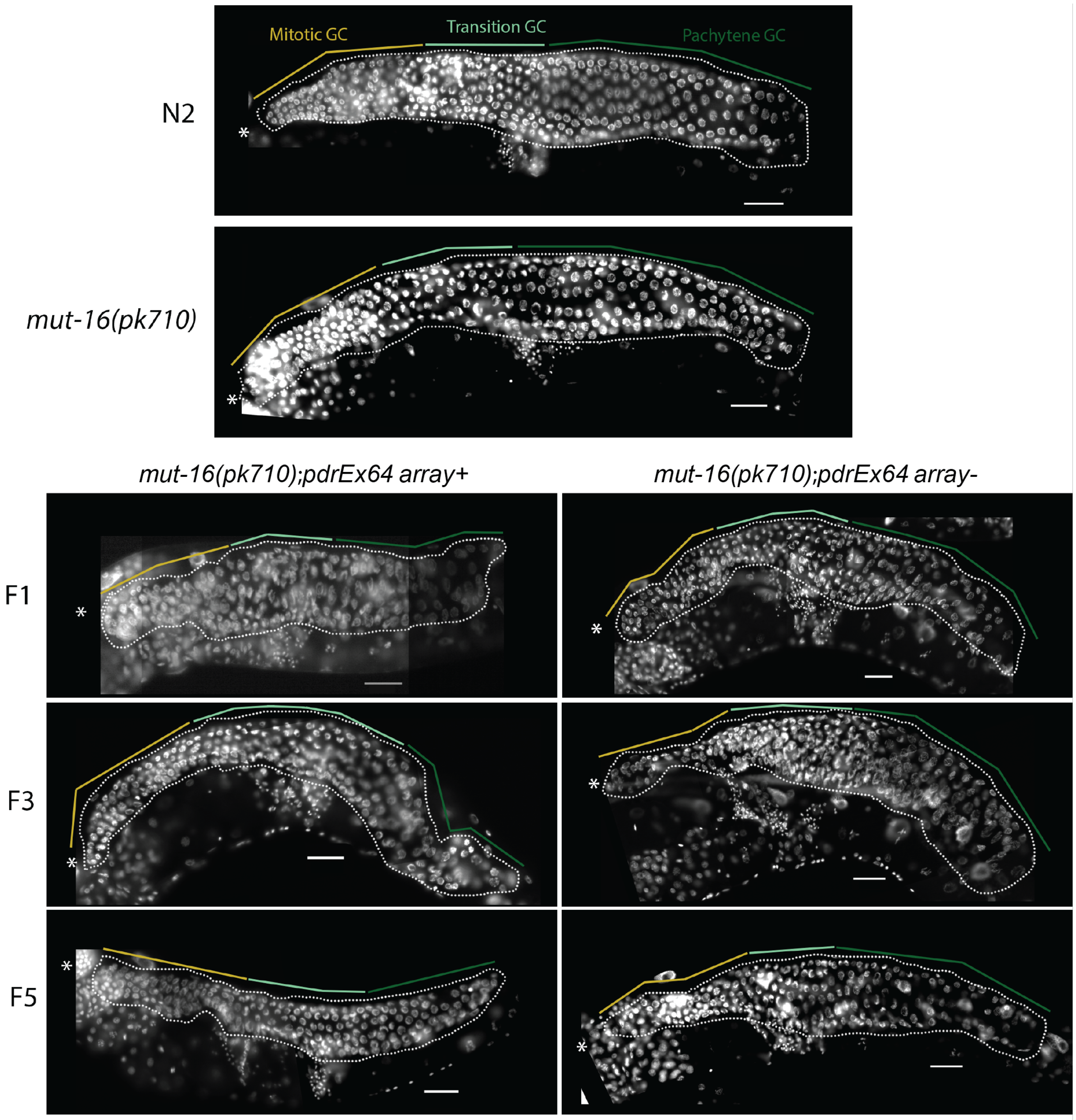

Figure S6. Pan-neuronal overexpression of *mut-16* correlates with decreased germ cell number. Figure shows representative images of DAPI-stained whole mount gonads of wild type N2, *mut-16(pk710),* and F1, F3, and F5 generations of the *mut-16(pk710) array+* and *array-* strains. The yellow, light green, and dark green lines indicate the mitotic, transition, and pachytene germline zones, respectively. The white dotted line demarks the part of the germline that includes the mitotic, leptotene, zygotene, and pachytene zones of young adult animals. Asterisks indicate the DTC. Scale bar is 25 µm.

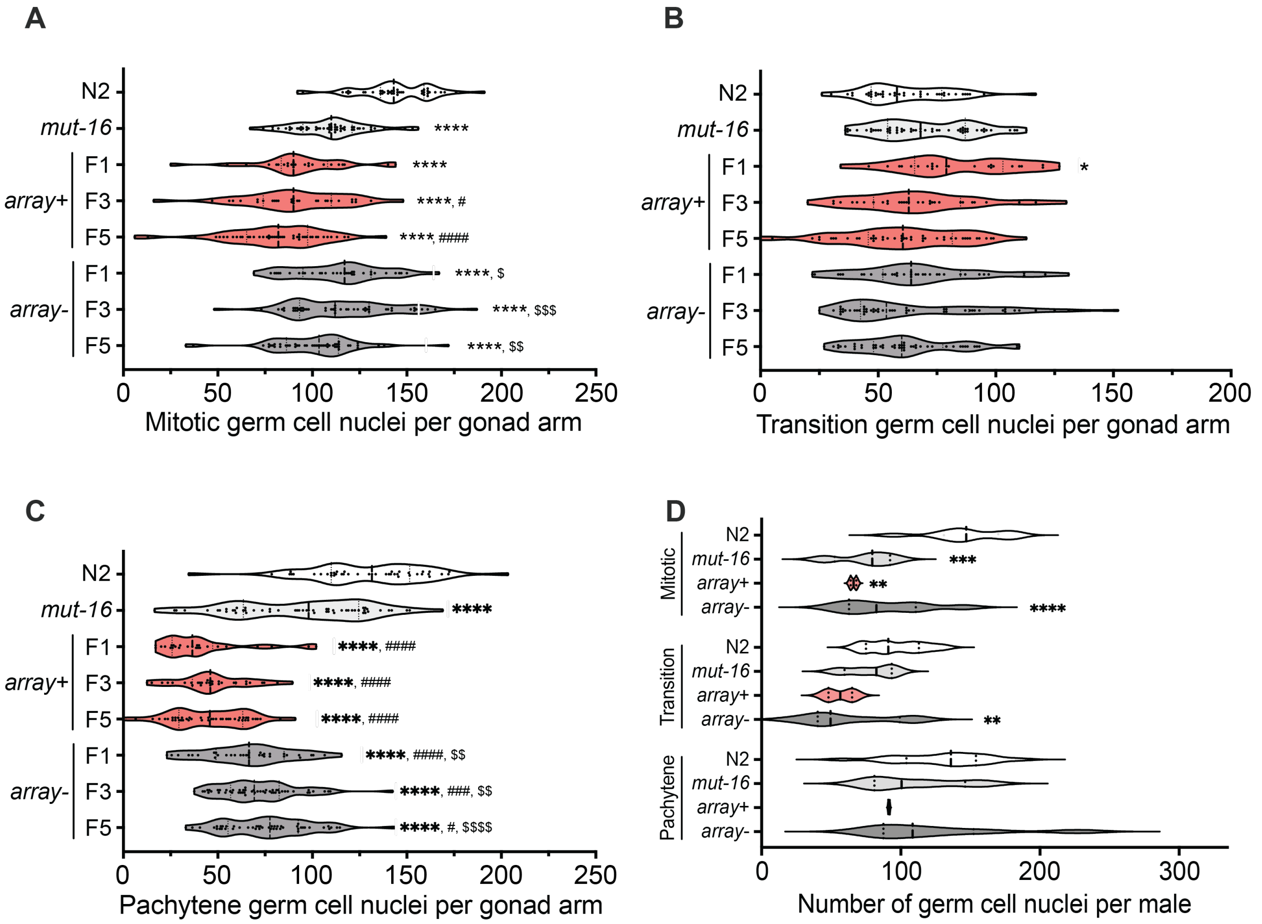

Figure S7. Pan-neuronal overexpression of *mut-16* is correlated with reduced germ cell numbers. (A, B, C) Violin plots represent the number of germ cell nuclei per hermaphrodite gonad arm for wild type N2, *mut-16(pk710),* and F1, F3, and F5 generations of *mut-16(pk710) array*+ and *array*- strains. The germ cell counts shown are for **(A)** mitotic, **(B)** transition, **(C)** and pachytene zones. n ≥ 25 gonad arms over 3 biologically independent trials. (D) Violin plots represent the number of germ cell nuclei per male for mitotic, transition, and pachytene zones in N2, *mut-16(pk710)*, and *mut-16 array*+ and *array*- strains. n ≥ 2. *, #, $ p < 0.05; **, ##, $$ p < 0.01; ***, ###, $$$ p < 0.001; ****, ####, $$$$ p < 0.0001; one-way ANOVA with Tukey's *post hoc* test * denotes a comparison to N2; # denotes a comparison to *mut-16(pk710),* $ denotes a comparison to *array*+ within the same generation. Additional data provided in Table S13.
